# Cohort identification of axial spondyloarthritis in a large healthcare dataset: current and future methods

**DOI:** 10.1186/s12891-018-2211-7

**Published:** 2018-09-05

**Authors:** Jessica A. Walsh, Shaobo Pei, Gopi K. Penmetsa, Jianwei Leng, Grant W. Cannon, Daniel O. Clegg, Brian C. Sauer

**Affiliations:** 1 Division of Rheumatology School of Medicine, 30 North 1900 East, Salt Lake City, UT 84132 USA; 2 George E. Wahlen Veteran Affairs Medical Center, 500 Foothill Boulevard, Salt Lake City, UT 84148 USA

**Keywords:** Spondyloarthropathy, Ankylosing spondylitis, Databases, Health services research

## Abstract

**Background:**

Big data research is important for studying uncommon diseases in real-world settings. Most big data studies in axial spondyloarthritis (axSpA) have been limited to populations identified with billing codes for ankylosing spondylitis (AS). axSpA is a more inclusive concept, and reliance on AS codes does not produce a comprehensive axSpA study population. The first objective was to describe our process for establishing an appropriate sample of patients with and without axSpA for developing accurate axSpA identification methods. The second objective was to determine the classification performance of AS billing codes against the chart-reviewed axSpA reference standard.

**Methods:**

Veteran Health Affairs clinical and administrative data, between January 2005 and June 2015, were used to randomly select patients with clinical phenotypes that represented high, moderate, and low likelihoods of an axSpA diagnosis. With chart review, the sampled patients were classified as Yes axSpA, No axSpA or Uncertain axSpA, and these classification assignments were used as the reference standard for determining the positive predictive value (PPV) and sensitivity of AS ICD-9 codes for axSpA.

**Results:**

Six hundred patients were classified as Yes axSpA (26.8%), No axSpA (68.3%), or Uncertain axSpA (4.8%). The PPV and sensitivity of an AS ICD-9 code for axSpA were 83.3% and 57.3%, respectively.

**Conclusions:**

Standard methods of identifying axSpA patients in a large dataset lacked sensitivity. An appropriate sample of patients with and without axSpA was established and characterized for developing novel axSpA identification methods that are anticipated to enable previously impractical big data research.

## Background

Big data research is necessary for studying uncommon diseases and outcomes in real-world settings [1]. Big data research is particularly important for spondyloarthritis (SpA), since concepts of SpA have broadened in recent years [2]. The axial SpA (axSpA) concept was introduced in 2009 [3], when it became apparent from advances in imaging and treatment that nearly one-half of patients with an axial inflammatory arthritis phenotype were excluded from traditional axSpA definitions (i.e. ankylosing spondylitis [AS]) [4]. Despite the growing recognition of more inclusive axSpA concepts, big data axSpA studies continue to be limited to the AS subtype [5–9], since there are no billing codes for non-AS subtypes. In more broadly defined axSpA populations, little is known about real-life outcomes, such as comorbidities, mortality, diagnostic and treatment patterns, health care utilization , and costs [10]. New methods of identifying axSpA patients are needed for a wide range of big data research in axSpA.

In order to develop and evaluate new axSpA identification methods, an appropriate sample of patients with and without axSpA is needed [11]. The ideal approach is to screen patients from the general population and classify them as having or not having axSpA. This approach is impractical for uncommon diseases, like axSpA, since tens of thousands of patients would need to be screened to identify a sufficient number of axSpA patients for research.

For feasibility purposes, patient populations are frequently enriched with patients at high risk of having the outcome of interest [12, 13]. For example, in an axSpA radiographic progression study, the population may consist of patients at elevated risk for structural disease progression (elevated C-Reactive Protein or baseline syndesmophytes) [14]. A disadvantage of this high-risk sampling approach is that the generalizability of the study results is limited by excluding lower risk patients (i.e. the results of the radiographic progression studies cannot be applied to axSpA patients without an elevated CRP or baseline syndesmophytes).

An alternative approach is to include both patients with high and low risk of disease. The sampled population is enriched for the disease of interest by including a greater percentage of patients at high risk than occurs in the general population. To improve generalizability, people at low risk are also required to be included in the sampled population. Compared to the distribution of high and low risk people in the general population, people at high risk for the disease are over-sampled and people at low risk are under-sampled [15]. This risk stratification sampling approach balances the advantages of feasibility with enrichment of high risk patients vs. generalizability with inclusion of low risk patients.

The first objective of this study was to describe our sampling strategy and chart-review process for establishing an appropriate sample of patients with and without axSpA for the future development of accurate axSpA identification methods for big data research. The second objective was to determine the classification performance of the International Classification of Diseases- Ninth Revision (ICD-9) code for AS against the chart-reviewed axSpA reference standard.

## Methods

### Design, setting, and data sources

This retrospective study used data from Veterans enrolled in the Veterans Health Administration (VHA). The data source was the Corporate Data Warehouse, a national repository of data from the VHA medical record system (VistA) and other VHA clinical and administrative systems [16]. The patient Integration Control Number was used to link patients across VHA stations. Data were housed and analyzed within the Veterans Affairs Informatics and Computing Infrastructure (VINCI) [17].

### Patient sampling strategy

The sampled population consisted of 600 randomly selected Veterans with conditions representing high (*n* = 200), moderate (*n* = 200), and low (*n* = 200) risk for axSpA, between January 1, 2005 to June 30, 2015. Structured Query Language (SQL) server newid function was used for randomization [18]. Veterans were considered by clinical experts to be at high risk for having axSpA if they had ≥1 AS ICD-9 code or a clinically available positive HLA-B27 result. Veterans with ICD-9 codes for sacroiliitis or a non-AS SpA subtypes (psoriatic arthritis, undifferentiated spondyloarthritis, reactive arthritis, and enteropathic arthritis) were categorized as moderate axSpA risk. The low risk category included patients with chronic back pain or other diseases that may mimic SpA [rheumatoid arthritis, diffuse idiopathic skeletal hyperostosis (DISH), crystal arthritis (gout and pseudogout), and other types of inflammatory arthritis (connective tissue disease, vasculitis, polymyalgia rheumatic, sarcoidosis, Paget’s disease)].

### AxSpA classification by chart review

Methods for classifying patients as having or not having axSpA occurred in multiple steps. First, clinical experts determined concepts that were expected to be useful for classification assignments. The concept categories included diagnostic language, disease features, laboratory results, and medications. Diagnostic language included statements affirming or negating the presence of SpA or an alternative diagnosis (i.e. “Mr. X has ank spond…”, “there is no evidence of spondyloarthritis”, or “her back pain is due to a herniated disk”. Disease features included language affirming or negating sacroiliitis, uveitis, enthesitis, inflammatory arthritis, dactylitis, psoriasis, inflammatory bowel disease, and syndesmophytes. Disease modifying anti-rheumatic drugs (DMARDs) included apremilast, leflunomide, methotrexate, sulfasalazine, adalimumab, certolizumab, etanercept, golimumab, infliximab, rituximab, and ustekinumab. Laboratory results included HLA-B27 positivity, elevated erythrocyte sedimentation rate, elevated C-reactive protein, rheumatoid factor, and anti-cyclic citrullinated protein.

Second, data were extracted for the 600 sampled patients. Four types of data were included: provider notes, imaging reports, laboratory results, and medications. For provider notes, all Text Integration Utility notes from primary care, rheumatology, orthopedics, gastroenterology, dermatology, ophthalmology, physical medicine and rehabilitation, pain clinics, geriatrics, emergency medicine, urgent care, and podiatry were extracted. Imaging reports were extracted with note titles indicating inclusion of a joint (neck, shoulder, elbow, wrist, hand, finger, pelvis, sacroiliac joint, hip, spine, knee, ankle, feet, and toe). The laboratory results were extracted by their Logical Observation Identifiers Names and Codes (LOINC) [19]. Quality review and revisions were used to ensure correct mapping and to standardize laboratory values. DMARD exposure data were extracted for all DMARDs dispensed during the study period.

Third, annotation software (eHOST [20]) was adapted and applied to the 600 sampled patients. A customized user interface was built for eHOST that enabled reviewers to efficiently view the extracted provider notes, imaging, laboratory results, and medications on a single screen, for the purpose of making patient-level classifications. Data were extracted and displayed in a manner that maintained the sequential nature and prioritization of the relevant documents. Annotation functions were designed for the reviewers to highlight and annotate the sections of text that were used to make classification decisions. Classification categories for axSpA status included Yes axSpA, No axSpA, and Uncertain axSpA. The uncertain category was assigned to patients with conflicting information or an axSpA diagnosis without additional information to support an axSpA diagnosis.

Two rheumatologists (JAW and GKP) independently annotated and classified the sampled patients. Classification guidelines were developed and revised. The protocol required reviewers, at a minimum, to assess specific types of documents (rheumatology consults and most recent rheumatology note, all articular imaging reports, dermatology notes, etc.). Additionally, the eHOST software was programmed to pre-annotate (highlight) every mention of terms relevant to axSpA (ankylosing spondylitis, spondyloarthr*, iritis, uveitis, dactylitis, enthesitis, erosion, *B27, etc.) in each document. After completing the initial annotation of the required documents (without pre-annotation), the reviewers annotated the pre-annotated terms that were not captured with the chart reviewers' previous annotation, to minimize the risk of overlooking data relevant to classification assignments. Both reviewers completed chart review classifications in batches of 20 until inter-rater agreement exceeded 85%. Discrepancies were adjudicated. After the 85% inter-rater agreement goal was achieved, the remainder of the sampled population was classified by a single reviewer.

### Characterizing the chart-reviewed population and evaluating AS ICD-9 codes

The demographics and health care utilization of the sampled patients were described in three groups: Yes axSpA, No axSpA, and Uncertain axSpA. Health care utilization was measured with duration of active VA system use during the study period (time between initial and most recent encounter with a provider or medication dispensation) and mean number of provider visits per year. The PPV of an AS ICD-9 code for axSpA was calculated in all patients with ≥1 ICD-9 code for AS [21]. The sensitivity of an AS ICD-9 code for axSpA was calculated in the subset who were *not* specifically selected to the sampled population because of an AS ICD-9 code. For PPV and sensitivity calculations, patients with sufficient evidence of axSpA (Yes axSpA) were compared to patients with insufficient evidence of axSpA (No axSpA and Uncertain axSpA). For confidence intervals, exact binomial confidence intervals were used for categorical variables and normal approximation was used for continuous variables.

## Results

### Patient population

During the study period, 9,803,429 Veterans participated in the VHA system. Patients eligible for selection into the sampled population included Veterans with ICD-9 codes for specific diseases or a laboratory result that placed them at high, moderate and low risk for axSpA (Table 1). Six hundred Veterans were randomly selected, including 0.83% of Veterans from the high risk stratum (*n* = 200), 0.25% from the moderate risk stratum (*n* = 200), and 0.01% from the low risk stratum (*n* = 200). Among the 600 sampled Veterans, 162 (27.0%) were classified as Yes axSpA, 409 (68.2%) were classified as No axSpA, and 29 (4.8%) were classified as Uncertain axSpA (Table 2). Within the group selected from the high risk stratum, 87% with an AS ICD-9 code were classified as Yes axSpA, while 38% of patients with a clinically available positive HLA-B27 result were classified as Yes axSpA. In the moderate risk groups, 27% with an ICD-9 code for a non-AS SpA subtype were classified as Yes axSpA and 7% with a sacroiliitis ICD-9 code were classified as Yes axSpA. In the low risk category, 2% of patients with an ICD-9 code for a SpA mimic were classified as Yes axSpA, and 1% of patients with ICD-9 codes chronic back pain were classified as Yes axSpA. The demographics and health care utilization patterns of the sampled population were similar between the Yes axSpA group and the No axSpA group, with the exception of younger age (56.2 vs. 59.9) and higher percentage of males (95.7% vs. 88.8%) in the Yes axSpA group (Table 3).

**Table 1:** Selection of patients sampled for the chart review population

Subgroups	Subgroup Criteria (ICD-9 or laboratory data)	No. of Veterans	No. of Veterans selected to chart review population	% from each risk stratum selected to the chart review population (95% CI)
High risk for axSpA				
Ankylosing spondylitis	720.0	15,862	100	0.83 (0.72–0.96)
HLA-B27 positivity	positive B27 test result	8168	100	
Moderate risk for axSpA				
Sacroiliitis	720.2	50,603	100	0.25 (0.21–0.28)
SpA subtype other than AS			100^a^	
Spondyloarthritis NOS	720.8× and/or 720.9×	6319		
Reactive arthritis	711.x and/or 99.3	1072		
Psoriatic arthritis	696.0	22,625		
Enteropathic arthritis	713.1 AND either 555.x OR 556.x	521		
Low risk of axSpA				
Chronic back pain	(≥2 ICD-9 codes for back pain ≥3 months apart [724.1, 724.2, 724.5])	2,069,644	100	0.01 (0.01–0.01)
Non-SpA rheumatologic disease			100^a^	
DISH	721.6	2963		
Crystal arthritis	274.x and/or 712.x	675,799		
Rheumatoid arthritis	714.x	143,620		
Other inflammatory arthritis	CTD (710.x), vasculitis (273.2, 446.0, 446.4, 446.5, 446.7), PMR (725), Paget’s (731.0), sarcoidosis (135)	135,608		

^a^25 patients from each subcategory of spondyloarthritis NOS, reactive arthritis, psoriatic arthritis, enteropathic arthritis, DISH, crystal arthritis, rheumatoid arthritis, and other inflammatory arthritis

**Table 2:** AxSpA classification by chart review

	All	High risk for axSpA		Moderate risk for axSpA		Low risk for axSpA	
	No. [%] (95% CI) *n* = 600	AS No. (95% CI) *n* = 100	HLA-B27+ No. (95% CI) *n* = 100	Non-AS SpA subtype No. (95% CI) *n* = 100	Sacroiliitis No. (95% CI) *n* = 100	SpA mimics No. (95% CI) *n* = 100	Chronic back pain No. (95% CI) *n* = 100
Yes AxSpA	162 [27.0] (23.5–30.7)	87 (78.8–92.9)	38 (28.5–48.3)	27 (18.6–36.8)	7 (2.9–13.9)	2 (0.2–7.0)	1 (0.0–5.5)
No AxSpA	409 [68.2] (64.3–71.9)	4 (1.1–9.9)	57 (46.7–66.9)	63 (52.8–72.4)	89 (81.2–94.4)	97 (91.5–99.4)	99 (94.6–100.0)
Uncertain AxSpA	29 [4.8] (3.3–6.9)	9 (4.2–16.4)	5 (1.6–11.3)	10 (4.9–17.6)	4 (1.1–9.9)	1 (0.0–5.5)	0 (0.0–3.6)

*No.* number, *CI* confidence interval

**Table 3:** Patient characteristics and health care utilization in chart review population

	Yes axSpA (*n* = 162)			No axSpA (*n* = 409)			Uncertain axSpA (*n* = 29)		
	No./Mean	SD/%	95% CI	No./Mean	SD/%	95% CI	No./Mean	SD/%	95% CI
Age	56.2	13.5	54.1, 58.3	60.0	13.2	58.7, 61.2	58.1	14.3	52.9, 63.3
Gender (Male)	155	95.7	91.3, 98.3	363	88.8	85.3, 91.7	28	96.6	82.2, 99.9
Race									
White	128	79.0	71.9, 85.0	305	74.6	70.1, 78.7	22	75.9	56.5, 89.7
Black	18	11.1	6.7, 17.0	63	15.4	12.0, 19.3	4	13.8	3.9, 31.7
Other	2	1.2	0.2, 4.4	6	1.5	0.5, 3.2	0	0.0	0.0, 11.9
Unknown	14	8.6	4.8, 14.1	35	8.6	6.0, 11.7	3	10.3	2.2, 27.4
Ethnicity									
Non-Hispanic	144	88.9	83.0, 93.3	367	89.7	86.4, 92.5	27	93.1	77.2, 99.2
Hispanic	7	4.3	1.8, 8.7	19	4.6	2.8, 7.2	2	6.9	0.9, 22.8
Unknown	11	6.8	3.4, 11.8	23	5.6	3.6, 8.3	0	0.0	0.0, 11.9
Geographic region									
Southeast	57	35.2	27.9, 43.1	153	37.4	32.7, 42.3	14	48.3	29.5, 67.5
North Atlantic	35	21.6	15.5, 28.7	84	20.5	16.7, 24.8	7	24.1	10.3, 43.5
Midwest	30	18.5	12.9, 25.4	68	16.6	13.2, 20.6	2	6.9	0.9, 22.8
Continental	22	13.6	8.7, 19.8	58	14.2	11.0, 17.9	4	13.8	3.9, 31.7
Pacific	18	11.1	6.7, 17.0	46	11.2	8.4, 14.7	2	6.9	0.9, 22.8
Duration of active VA system use during study period (years)	9.3	2.0	9.0, 9.6	8.9	2.4	8.7, 9.2	9.0	2.2	8.2, 9.8
#Provider visits/ year during active system use period	43.6	39.3	37.5, 49.6	45.9	42.6	41.7, 50.0	25.5	27.3	15.6, 35.5

*No.* number, *VA* Veteran Affairs, *CI* confidence interval

### Performance of AS diagnosis codes for classifying axSpA

**Fig. 1:**
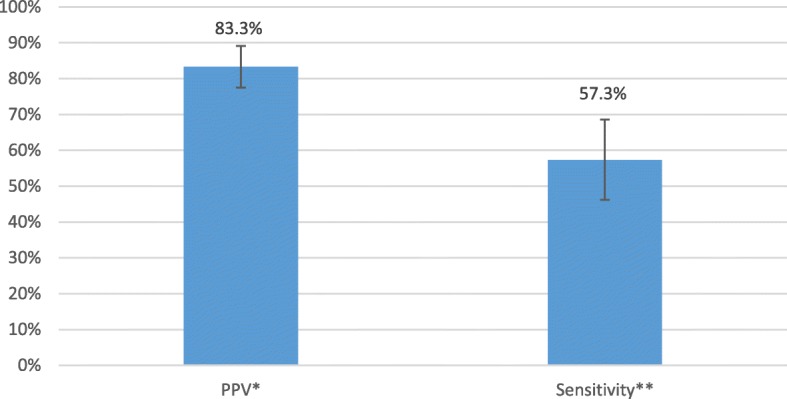
Positive predictive value and sensitivity of ankylosing spondylitis ICD-9 code for AxSpA. PPV = positive predictive value. *n = 156 Veterans with an AS ICD-9 code. **n = 75 Veterans with AxSpA who were *not* selected to the chart review sample specifically for an AS ICD-9 code

Among the 156 axSpA patients with an AS ICD-9 code, the PPV of the AS ICD-9 code for axSpA was 83.3% (Fig. 1). Within the 75 axSpA patients who were *not* specifically selected to into the sampled population because of an AS ICD-9 code, the sensitivity of an AS ICD-9 code for axSpA was 57.3%.

## Discussion

We established and characterized an appropriate sample of patients with and without axSpA for developing novel axSpA identification methods and for evaluating AS billing codes in axSpA patients. This population is enriched with axSpA patients and is representative of more generalizable disease states (i.e. chronic back pain). Additionally, patients were included with diseases that may mimic axSpA (DISH, peripheral psoriatic arthritis, crystal arthritis, etc.) to maximize the ability of axSpA identification methods to differentiate between axSpA and axSpA mimics. These sampled patients will be used to identify and prioritize data that differentiate between patients with and without axSpA, for the development of novel axSpA classification algorithms.

The evaluation of the AS ICD-9 code demonstrated a reasonably high PPV for axSpA (83.3%). This is similar to the PPV of ≥1 AS ICD-9 code for AS reported in a Veteran population attending rheumatology clinics (83%) [22]. However, the PPV estimates in both of these studies are likely overestimated compared to the PPV in the general population, since these population were enriched with AS patients, and PPV estimates increase when the prevalence of the underlying condition increases.

The sensitivity of the AS ICD-9 code for axSpA was low (57%). Not all patients with axSpA have AS, since axSpA also includes non-AS subtypes. However, it is likely that providers used the AS ICD-9 code as a proxy for non-AS axSpA subtypes, since there are no billing codes for non-AS axSpA subtypes. While precise usage patterns of AS billing codes remain unknown, the lack of an AS ICD-9 code in 43% of axSpA patients over the 10.5 year study period, demonstrated that nearly one-half of axSpA patients were not identifiable with the only ICD-9 billing code that indicates that presence of inflammatory axial arthritis.

Strengths of this study include the large sample size and access to a medical record system that enabled consistent data capture across VHA sites throughout the United States. Furthermore, the chart review process was feasible for comprehensive review of multiple types of data for several hundred patients over a period exceeding 10 years. Another strength was the clinical expertise of the chart reviewers. As rheumatologists specializing in SpA within the VA system, both reviewers are experienced with the intricacies of axSpA patient care and documentation within the VA system.

This study was limited by the inability of chart reviewers to directly interact with patients or access digital radiologic images (radiology reports were reviewed). Thus, classification decisions had to be made with incomplete and otherwise imperfect data. These data limitations inevitably led to classification errors, particularly in patients with sparse data. Since patients with early or mild axSpA are expected to have fewer encounters than established and severe axSpA patients, the classification process was likely more accurate with established and more severe axSpA than early and milder axSpA [23]. Another limitation is that ICD-10 codes for axSpA were not evaluated, since the study period preceded the implementation of ICD-10 codes. It is anticipated that ICD-10 codes will be similarly limited with axSpA cohort identification, since ICD-10 codes do not include non-AS axSpA subtypes.

## Conclusion

There is an unmet need for accurate methods of identifying axSpA patients in large datasets. We established and characterized an appropriate sample of patients for developing axSpA identification methods that are anticipated to enable a wide array of previously impractical big data studies in axSpA.

## Abbreviations

AS: Ankylosing spondylitis
axSpA: Axial spondyloarthritis
DISH: Diffuse idiopathic skeletal hyperostosis
ICD: International Classification of Diseases
PPV: Positive predictive value
SpA: Spondyloarthritis
VHA: Veterans Health Administration
